# Management of Infantile Spasms During the COVID-19 Pandemic

**DOI:** 10.1177/0883073820933739

**Published:** 2020-06-23

**Authors:** Zachary M. Grinspan, John R. Mytinger, Fiona M. Baumer, Michael A. Ciliberto, Bruce H. Cohen, Dennis J. Dlugos, Chellamani Harini, Shaun A. Hussain, Sucheta M. Joshi, Cynthia G. Keator, Kelly G. Knupp, Patricia E. McGoldrick, Katherine C. Nickels, Jun T. Park, Archana Pasupuleti, Anup D. Patel, Asim M. Shahid, Renee A. Shellhaas, Daniel W. Shrey, Rani K. Singh, Steven M. Wolf, Elissa G. Yozawitz, Christopher J. Yuskaitis, Jeff L. Waugh, Phillip L. Pearl

**Affiliations:** 1 5922 Weill Cornell Medicine, New York, NY, USA; 2 Nationwide Children’s Hospital, Columbus, OH, USA; 3 6429 Stanford University School of Medicine, Palo Alto, CA, USA; 4 4083 University of Iowa Hospitals, Iowa City, IA, USA; 5 Children’s Hospital Medical Center of Akron, Akron, OH, USA; 6 Children’s Hospital of Philadelphia, Philadelphia, PA, USA; 7 Department of Neurology, 1862 Boston Children’s Hospital, Boston, MA, USA; 8 155697 University of California Los Angeles Mattel Children’s Hospital, Los Angeles, CA, USA; 9 University of Michigan Pediatric Neurology, Ann Arbor, MI, USA; 10 Cook Children’s Medical Center, Fort Worth, TX, USA; 11 Children’s Hospital Colorado, Aurora, CO, USA; 12 Boston Children’s Health Physicians, Hartsdale, NY, USA; 13 Mayo Clinic, Rochester, MN, USA; 14 159284 University Hospitals Rainbow Babies & Children’s Hospital, Cleveland, OH, USA; 15 Children’s National Hospital, Washington, DC, USA; 16 Children’s Hospital of Orange County, Orange, CA, USA; 17 Levine Children’s Hospital at Atrium Health System, Charlotte, NC, USA; 18 550033 Montefiore Medical System, Bronx, NY, USA; 19 1862 University of Texas Southwestern Medical Center Southwestern, Dallas, TX, USA

**Keywords:** infantile spasms, West syndrome, antiseizure drugs, electroencephalography, EEG

## Abstract

Circumstances of the COVID-19 pandemic have mandated a change to standard management of infantile spasms. On April 6, 2020, the Child Neurology Society issued an online statement of immediate recommendations to streamline diagnosis and treatment of infantile spasms with utilization of telemedicine, outpatient studies, and selection of first-line oral therapies as initial treatment. The rationale for the recommendations and specific guidance including follow-up assessment are provided in this manuscript. These recommendations are indicated as enduring if intended to outlast the pandemic, and limited if intended only for the pandemic health care crisis but may be applicable to future disruptions of health care delivery.

Infantile spasms are an early-life epilepsy syndrome and epileptic encephalopathy characterized by epileptic spasms that typically occur in clusters. The incidence is 1 per 2400 to 5500 live births,^1-7^ roughly similar to the incidence of cystic fibrosis,^8^ tetralogy of Fallot,^9^ and type 1 diabetes.^10^ The age of onset is usually between 2 and 9 months, though infantile spasms can present up to age 2 years.^11^ Rapid diagnosis and treatment of infantile spasms is a key priority for pediatric neurologists because timely delivery of effective treatment improves the likelihood of resolution of epileptic spasms and improves cognitive outcomes.^12-15^

Developmental outcomes at 18 months depend strongly on pre-existing risk of developmental impairment, pre-existing epilepsy at the time of diagnosis, etiology, and treatment response.^14,15^ For example, in infants with a history of hypoxic ischemic encephalopathy, infantile spasms is a marker of injury severity and associated with significant neurodevelopmental disability.^16-18^ In contrast, in infants with normal brain magnetic resonance imaging (MRI), normal prior development, no prior epilepsy, no clear etiology, and brisk response to treatment (within 7 days), a normal neurodevelopmental trajectory is common.^12,14,19^

In the United States, the initial evaluation for infantile spasms typically includes an inpatient admission for video electroencephalography (EEG).^20^ The hospital admission allows the clinical team to confirm the diagnosis, begin treatment, investigate the etiology (ie, via brain MRI and other testing), and provide counseling to the family. When adrenocorticotropin (ACTH) is prescribed, the inpatient admission also allows the clinical team to coordinate training for caregivers on how to administer injections, obtain health insurance authorization, and arrange home delivery. When hormonal treatments are prescribed (ACTH or oral corticosteroids), infants are often monitored during therapy for hypertension and glucosuria. Modern regimens are typically brief (2 weeks at a high dose followed by a 2-week taper) because prolonged courses of ACTH or prednisolone risk immunosuppression and severe hypertension.^21^

The COVID-19 pandemic has strained traditional health care delivery. At the time of writing (April 2020), COVID-19 infections are common among patients admitted to hospitals, and many pediatric wards are occupied by sick adults. Local, regional, and national governments worldwide have instituted social distancing measures to reduce community transmission, including closing businesses and public spaces, and ordering the general public to stay at home. Every visit to a health system puts children and their families at risk for infection, and increases the risk of inadvertent exposure to health care providers. The risk of exposure is magnified due to transmission by presymptomatic infected COVID-19 carriers^22^ and airborne (ie, aerosol) transmission of COVID-19.^23,24^ Although COVID-19 infections are milder in pediatric populations, infants and immunocompromised individuals have the most severe disease among infected children.^25,26^

In recognition of the need to balance rapid evaluation of infantile spasms with the public health demands of a pandemic, a group of pediatric epilepsy experts in the United States drafted recommendations for the diagnosis and treatment of infantile spasms in the COVID-19 era.

The recommendations are endorsed by the Child Neurology Society, the North American–based professional organization of child neurologists established in 1972.^27^ The text was drafted in collaboration with the Pediatric Epilepsy Research Consortium, a group of 54 US academic pediatric epilepsy centers committed to collaborative, collegial, practice changing research.^28^ The authors drafted, edited, and finalized an initial statement via e-mail, telephone, and teleconference during the week of March 30 to April 6, 2020. These were published online through the Child Neurology Society website on April 6, 2020.^29^

This manuscript reproduces the recommendations, with minor modifications, supplemented with references and rationale. We have also indicated if each recommendation is *limited* (ie, intended only for the COVID-19 pandemic health care crisis) or *enduring* (ie, intended to outlast the pandemic). The recommendations are targeted to the current pandemic but may be applicable to future disruptions of health care delivery.

## Recommendations

### Diagnosis of New-Onset Infantile Spasms

*The initial clinical visit for suspected infantile spasms may be performed by telemedicine or video conference. [Enduring]*

In-person access to child neurology clinical expertise is uneven in the United States.^30,31^ Telemedicine allows clinicians to evaluate children from afar. Prior to the COVID-19 pandemic, growing experience suggested telemedicine was a feasible and effective way to expand access to neurology care for children.^32-36^ For infantile spasms, clinical decision-making relies on the infant’s medical and neurologic history and the characteristics of the events -- these can be evaluated remotely.

*Instruct parents to obtain and share video recordings of several consecutive typical events. Review the videos prior to or during the initial clinical encounter. [Enduring]*

Given that 92% of US adults aged 30-49 years own a smartphone,^37^ it is reasonable to ask a parent or caregiver to obtain home video recording of the events. Individual epileptic spasms are typically flexor movements (though they can be extensor) of the arms, legs, and/or neck, usually lasting less than 2 seconds.^38^ The movement lasts longer than myoclonus, but briefer than a tonic seizure. Of key importance to the diagnosis of infantile spasms, the epileptic spasms usually occur at regular intervals (every 3-30 seconds)^39^ within clusters lasting several minutes. Parents and caregivers should be requested to capture at least 3 events, including the interval between events (intervals less than 3 seconds are essentially never epileptic spasms). The entire body and face should be within camera view along with adequate lighting. If possible, the recordings should include the infant in multiple positions (eg supine and seated) and multiple states (eg awake and asleep).

*Ask about light spots on the skin and/or perform skin examination (ie, as a possible indicator of tuberous sclerosis complex.) [Enduring]*

Tuberous sclerosis complex is a common cause of infantile spasms.^40,41^ Two in five infants with tuberous sclerosis complex will develop infantile spasms,^42^ and in some cases, infantile spasms are the presenting symptom.^43^ Hypomelanotic macules are present in 90% of patients with tuberous sclerosis complex and are typically present at birth or during infancy.^44^ Subtle findings may be missed via telemedicine; thus, a full dermatologic examination with Wood’s lamp should be performed when possible. Of important note, tuberous sclerosis complex is a clinical diagnosis based on examination and imaging and does not require genetic confirmation.^44^

*EEG confirmation is strongly encouraged and should include at least 1 sleep-wake cycle. [Enduring]*

There are many mimics of infantile spasms, including gastroesophageal reflux, benign shuddering attacks, benign myoclonus of infancy, and other seizure types (eg, myoclonic or tonic seizures).^45^ Therefore, EEG is foundational to establish the diagnosis of infantile spasms. EEG typically shows epileptiform abnormalities potentiated by sleep.^46^ Of important note, hypsarrhythmia is not required for diagnosis. Hypsarrhythmia is absent in as many as one-quarter of cases,^7,47,48^ and its presence at diagnosis does not predict treatment response.^47^ If the EEG background is normal but clinical concern is high, then a longer video recording that captures the events should be obtained.

*During the COVID-19 pandemic, outpatient EEG is preferred over inpatient admission. [Limited]*

Inpatient care allows rapid coordination of video EEG, brain MRI, laboratory testing, treatment initiation (or coordination), and caregiver education, as well as access to first-line medications like ACTH and vigabatrin, which are not available in commercial outpatient pharmacies. However, the need for inpatient care is not driven by a meaningful risk of cardiorespiratory instability or acute neurologic decompensation. Thus, the benefits of inpatient care coordination must be balanced with the risk of exposure to infected individuals, such as health care workers or other admitted patients and their families.

*After diagnosis, if the etiology is uncertain, obtain a brain MRI (urgent; within 1-3 days) and perform genetic testing (expedited). [Enduring]*

If the etiology of infantile spasms is not clear after thorough history and physical examination, a brain MRI is crucial to the etiological workup, as it can affect treatment choices. Two examples follow. First, the diagnosis of tuberous sclerosis complex may be made by brain MRI, because skin lesions can be subtle, and affected children may first come to medical attention because of infantile spasms.^43^ The diagnosis affects treatment selection, as tuberous sclerosis complex is preferentially treated with vigabatrin (rather than hormonal therapy).^49,50^ Second, when a structural brain lesion is identified, an epilepsy surgery evaluation can be expedited if standard medical treatment fails.^51,52^

Genetic testing is also a high-yield priority early in the diagnostic workup. A pathogenic genetic abnormality is found in nearly half of those tested,^11^ including in 24% of those where the initial history, examination, and MRI do not identify a cause.^53^ A comprehensive epilepsy gene panel is generally recommended as the first-line test at this time, and microarray may be considered in the presence of dysmorphism or other organ system involvement.

### Treatment

*Select from among ACTH (adrenocorticotropic hormone), high-dose prednisolone (4-8 mg/kg/d), and vigabatrin, unless there are contraindications to all three. [Enduring]*

ACTH, prednisolone, and vigabatrin have the strongest evidence to support use as the first line for infantile spasms. This recommendation is backed by consensus statements, structured literature reviews, guidelines from national organizations, and quality measures, based on a synthesis of evidence that includes high-quality randomized controlled trials.^54-57^

*During the COVID-19 pandemic, initiation of high-dose oral prednisolone in the outpatient setting is the preferred initial treatment for infantile spasms for etiologies other than tuberous sclerosis complex. [Limited]*

Among the three recommended first-line therapies for infantile spasms, prednisolone has several advantages that make it preferable during the COVID-19 pandemic. It is inexpensive, readily available in commercial pharmacies, and orally administered. This is in contrast with ACTH and vigabatrin, which require special training of caregivers (ACTH, injections; vigabatrin, mixing of sachets), use of subspecialty pharmacies, and preapproval by insurance. High-dose prednisolone regimens (4-8 mg/kg/d) have better response rates than low dose prednisolone,^58^ though the optimal regimen has not been established. We provide two sample regimens, one used in European clinical trials^14,59^ and one used by many US programs^60,61^ (Table 1).

**Table 1.** Two High-Dose Prednisolone Regimens, for Infantile Spasms.

Days	Prednisolone Dose, **Weight Based** ^60,61^		
1-14	8 mg/kg/day divided 3 or 4 times a day (maximum 60mg/day)		
15-17	6 mg/kg/day divided 3 times a day		
18-20	4 mg/kg/day divided twice a day		
21-23	2 mg/kg/day once a day (in the morning)		
24-26	1 mg/kg/day once a day (in the morning)		
27-29	0.5 mg/kg/day once a day (in the morning)		
30	STOP		
Days	Prednisolone Dose, **Fixed Dose** ^14,59^		
1-7	10 mg 4 times daily		
	*Clinical spasms stop for 24 hours*		*Clinical spasms continue*
8-14	10 mg 4 times daily		20 mg 3 times daily
15-19	10 mg 3 times daily		10 mg 4 times daily
20-24	10 mg twice daily		
25-29	10 mg daily		
30	STOP		

Three additional comments deserve mention.

First, if evaluating infantile spasms in the inpatient setting, length of stay may be reduced (and COVID-19 risk minimized) by selection of prednisolone or vigabatrin over ACTH.^62^

Second, this limited recommendation is intended to help clinicians balance the risks of COVID-19 exposure with the provision of timely clinical care, and should not be used to justify denial of insurance coverage or payment for inpatient hospitalization, for use of ACTH, or for use of vigabatrin.

Third, some clinicians may elect to start prednisolone and then switch to ACTH or vigabatrin when available (and provide outpatient or home training), or add vigabatrin based on potential benefits of combination therapy.^14,63^

*For tuberous sclerosis complex, vigabatrin is preferred if immediately available. If access is delayed, while waiting for vigabatrin, initiate oral prednisolone and obtain an echocardiogram. [Enduring]*

Infantile spasms due to tuberous sclerosis complex respond particularly well to vigabatrin.^54-56^ However, access to medications from specialty pharmacies may be delayed during the COVID-19 pandemic, and early treatment of infantile spasms is essential. Prednisolone is widely available and can be easily started while waiting to obtain vigabatrin. ACTH has been reported to increase the size of cardiac rhabdomyomas^64,65^; thus, use of hormonal therapy in tuberous sclerosis complex should include echocardiography evaluation.

*Avoid non-standard therapy as the first treatment choice for infantile spasms (ie, avoid topiramate, ketogenic diet, etc.). [Enduring]*

Other anti-seizure medications and dietary therapies are not as efficacious as hormonal therapy or vigabatrin and should not be administered as initial therapy. In particular, though topiramate showed early promise,^66,67^ follow-up analyses have failed to show efficacy against infantile spasms.^68-72^ Zonisamide^73,74^ and levetiracetam^69^ also lack compelling evidence of efficacy. Ketogenic diet^75,76^ and valproic acid^50^ may have efficacy, but the evidence does not support their use as initial treatment for infantile spasms.

*For infants treated with prednisolone or ACTH, consider GI prophylaxis with a proton pump inhibitor or H2 blocker. [Enduring]*

Medications for prophylactic gastric acid suppression during the treatment of infantile spasms is common practice,^49^ and their overall efficacy in children is well established. Three commonly used and readily available regimens^i^ include:

- Famotidine 0.5 mg/kg/dose twice daily
- Omeprazole 1 mg/kg daily
- Lansoprazole 15 mg once daily (if <30 kg)

*For treatment with prednisolone or ACTH, write a single prescription that includes both 2 weeks at a high dose and a 2-week taper. [Enduring]*

The authors have cared for children for whom high-dose hormonal therapy was initiated, but there was no follow-up at 2 weeks to initiate the taper. Therefore, the taper should be specified from the onset of treatment to prevent medication over-exposure or abrupt cessation.

### Follow Up After Initial Diagnosis and Treatment of Infantile Spasms

*Follow infants closely via telehealth, video conference, or phone calls, at least weekly. If using hormonal therapy, measure blood pressure at least weekly during treatment. Laboratory testing should be used sparingly, unless there are clinical indications that suggest a specific need for more than routine monitoring. [Limited]*

Clinically important laboratory abnormalities are often accompanied by clinical signs (ie, lethargy or reduced feeding), but hypertension may be initially asymptomatic. Hormonal therapy of infantile spasms commonly leads to hypertension and, in some cases, cardiomegaly.^59,77^ When hypertension occurs, it typically begins within a week of therapy, though there may be a delayed response after 3 weeks.^21,77^ Hypertension typically resolves after medication discontinuation.^77^ In older series, treatment-related deaths occurred as a result of infection and hemorrhage.^21^ Large European and US cohorts of infantile spasms from the 2000s and 2010s^40,59,63^ did not report treatment-related deaths, though case series data suggest they still occur.^78^

*Assess treatment response via telehealth or video conference at least weekly. After 7-10 days, if clinical infantile spasms continue, consider adding or modifying treatment without confirmatory EEG. If clinical infantile spasms have resolved, or if the caregiver is uncertain, a repeat EEG, including at least one sleep-wake cycle, is strongly encouraged. Outpatient EEG is preferred over inpatient admission. [Limited]*

Clinical resolution of epileptic spasms per caregiver report can be misleading, as spasms can become less obvious as a partial response to treatment.^79^ Caregivers may underestimate the number of spasms or miss subtle or single isolated spasms.^54^ In addition, EEG abnormalities may persist and are typically most obvious during sleep.^46^ Caregiver-reported clinical resolution can be confirmed by an EEG (ideally with video) that captures at least one sleep-wake cycle, and shows neither epileptic spasms nor hypsarrhythmia.

## Note

i. Ranitidine was often prescribed in the recent past; it is not currently available because of concerns about a contaminant that may increase cancer risk (https://www.fda.gov/news-events/press-announcements/fda-requests-removal-all-ranitidine-products-zantac-market).
